# Exercise-Induced Mitohormesis for the Maintenance of Skeletal Muscle and Healthspan Extension

**DOI:** 10.3390/sports7070170

**Published:** 2019-07-11

**Authors:** Robert V. Musci, Karyn L. Hamilton, Melissa A. Linden

**Affiliations:** Department of Health and Exercise Science, Colorado State University, Fort Collins, CO 80523, USA

**Keywords:** redox homeostasis, mitohormesis, mitochondrial function, skeletal muscle, aging, sarcopenia, exercise, healthspan

## Abstract

Oxidative damage is one mechanism linking aging with chronic diseases including the progressive loss of skeletal muscle mass and function called sarcopenia. Thus, mitigating oxidative damage is a potential avenue to prevent or delay the onset of chronic disease and/or extend healthspan. Mitochondrial hormesis (mitohormesis) occurs when acute exposure to stress stimulates adaptive mitochondrial responses that improve mitochondrial function and resistance to stress. For example, an acute oxidative stress via mitochondrial superoxide production stimulates the activation of endogenous antioxidant gene transcription regulated by the redox sensitive transcription factor Nrf2, resulting in an adaptive hormetic response. In addition, acute stresses such as aerobic exercise stimulate the expansion of skeletal muscle mitochondria (i.e., mitochondrial biogenesis), constituting a mitohormetic response that protects from sarcopenia through a variety of mechanisms. This review summarized the effects of age-related declines in mitochondrial and redox homeostasis on skeletal muscle protein homeostasis and highlights the mitohormetic mechanisms by which aerobic exercise mitigates these age-related declines and maintains function. We discussed the potential efficacy of targeting the Nrf2 signaling pathway, which partially mediates adaptation to aerobic exercise, to restore mitochondrial and skeletal muscle function. Finally, we highlight knowledge gaps related to improving redox signaling and make recommendations for future research.

## 1. Introduction

In the next half century, the world’s population of individuals aged over 65 years will significantly increase. The global population of individuals over the age of 65 will likely double from 6.9% to 12.0% by 2030 [1]. Specifically, in Europe, projections suggest that individuals over the age of 65 years will comprise of 28% of the population by 2060, compared to 18% in 2013 [2]. Similarly, in the United States, 25.8% of the population will be above the age of 65 years by 2050 [3].

With the growing aging population, researchers and healthcare providers have shifted focus to extending healthspan (as opposed to the lifespan), or the period of time living free of age-related chronic diseases, such as diabetes, cardiovascular disease, and Alzheimer’s disease [4,5]. There are nine underlying mechanisms linking aging with chronic diseases that are highlighted in the review “Hallmarks of Aging [6].” Targeting these mechanisms to slow aging should mitigate the burden and duration of chronic diseases, expanding healthspan [7]. Often touted as a beneficial health behavior, exercise is somewhat underappreciated as a means to target many of the hallmarks of aging and slow age-related declines in function. However, there is growing appreciation that exercise, particularly aerobic, is an effective means to extend healthspan [8]. One of the suspected primary mechanisms by which aerobic exercise confers beneficial health adaptations is through mitohormesis. Mitohormesis is a term used to describe the response to an acute, sublethal stress, such as oxidative stress, that may temporarily impair or damage mitochondria, but ultimately leads to the activation of adaptive mechanisms that confer stress resistance and improve mitochondrial function [9]. In this review, we describe the age-related decline in mitochondrial function, the concomitant decline in redox homeostasis, and their combined deleterious effect on skeletal muscle function. We then discuss the mitohormetic effect of an acute bout of aerobic exercise as a means to improve redox homeostasis and mitochondrial function. In addition, we highlight the mitohormetic adaptations to lifelong aerobic exercise as a means to prevent age-related declines in mitochondrial function and redox homeostasis to maintain skeletal muscle health with age.

## 2. Age-Related Decline in Mitochondrial Function

While it is unclear if mitochondrial dysfunction is a cause or a consequence of aging, mitochondrial dysfunction is indeed a “hallmark” of aging [6]. Two key characteristics of mitochondrial dysfunction are decrements in ATP production and a concomitant increase in reactive oxygen species (ROS) production [10]. This review predominantly focuses on these two characteristics; however, it is important to note that there are several other components of mitochondrial function that are impaired with age (e.g., mitochondrial dynamics [11,12], calcium handling [13]). Some cross-sectional studies demonstrate that older adults generally have lower mitochondrial ATP production with chronological age in skeletal muscle [14,15,16], whilst others do not [17,18,19]. Interindividual variability, study design, and methods utilized to measure mitochondrial function likely contribute to these divergent findings. When covariates such as physical activity, fitness, and adiposity are controlled for, there is only a weak inverse correlation between age and mitochondrial function that accounts for less than 5% of variations observed in maximal oxidative capacity in mitochondria [20]. This relatively minimal contribution of age to mitochondrial dysfunction emphasizes that dysfunction is not entirely programmed into the aging process. Rather, it suggests that age-related mitochondrial dysfunction is modifiable through factors such as health behaviors (diet and exercise). Thus, there is potential for interventions to target mitochondrial dysfunction and prevent the age-related diseases that arise as a consequence of it.

## 3. Sarcopenia and Its Effect on Overall Health

Sarcopenia was originally characterized in 1989 by Dr. Irwin Rosenberg as the age-related loss of skeletal muscle mass [21]. Over the past three decades, this definition has evolved to include the age-related loss of skeletal muscle function [22]. Whilst there is debate on the value of returning to the strict definition of sarcopenia as loss of muscle mass and establishing new terminology to characterize age-related muscle dysfunction [23,24], the World Health Organization (WHO) recently established an ICD-10 code for sarcopenia defined as loss of skeletal muscle mass and function, which will certainly spur new research and treatment [25]. In this review, we use the term sarcopenia as defined by the WHO.

Skeletal muscle function (e.g., muscle strength, mass, and overall mobility [22]) has a significant impact on quality of life and overall health. Sarcopenia increases the risk of disability for men and women two- and three-fold, respectively, compared to non-sarcopenic individuals [26]. Longitudinal studies, such as the Health ABC study, also demonstrate that age-related muscle loss predicts the loss of strength and the incidence of disability [27,28]. Sarcopenia increases the risk of falls three-fold in adults above the age of 80 years [29]. Lower levels of muscle function and the increased risk of disability and frailty also hinders recovery from hospitalization, predisposing individuals to admission to nursing home facilities [30].

While the role of skeletal muscle in the maintenance of quality of life and mobility through aging is generally appreciated, skeletal muscle also plays a significant role in overall metabolic health and longevity. Over the past decade, there is greater acknowledgment of the role of skeletal muscle as being central to substrate metabolism and as an endocrine organ [31]. For example, given the role of skeletal muscle in glucose metabolism, there is growing speculation that sarcopenia could impair insulin sensitivity [32] and several prospective studies have indeed linked sarcopenia with increased incidence of type 2 diabetes [33,34,35]. Sarcopenia is associated with the increased risk of developing other chronic diseases such as cardiovascular disease, which could be mediated by increased inflammatory cytokines released by skeletal muscle [36,37,38]. In addition, skeletal muscle function predicts the survival rates of other diseases such as cancer [39]. While the potential mechanisms by which skeletal muscle and metabolic health contribute to healthspan continue to be elucidated, it is clear that maintaining skeletal muscle function imparts health beyond mobility.

## 4. Skeletal Muscle Mitochondrial Dysfunction and Sarcopenia

Sarcopenia is a multi-faceted syndrome with a multitude of contributing factors. The loss of skeletal muscle mass is perhaps one of the strongest factors linked to the loss of skeletal muscle function. Koopman and van Loon highlight that thigh muscle size explains 49% of the variability in maximal leg press strength [40]. Studies almost always demonstrate that older adults have both lower skeletal muscle mass and strength than younger adults [28,41,42,43,44]. However, it is important to acknowledge that even when controlling for skeletal muscle size, specific strength is still impaired with age [41]. These data indicate that other factors besides muscle mass contribute to overall strength and function, including energetics [45], muscle fiber type [42], innervation [46], redox signaling [47], and skeletal muscle proteome integrity [48,49]. These “other factors,” provide opportunities to discover complementary interventions to preserve skeletal muscle function with age, contributing to overall healthspan. Mitochondrial function plays a significant underlying role in many of these factors, including maintaining energetics [18], skeletal muscle innervation [50], proteome integrity (proteostasis) [51], and redox homeostasis [52]. The remainder of this review will focus on the role of mitohormesis in the maintenance of mitochondrial function and subsequently of skeletal muscle function.

Mitochondrial function has a significant effect on skeletal muscle function. The Baltimore Longitudinal Study on Aging, perhaps one of the most comprehensive longitudinal studies that follows muscle function, aerobic capacity, and mitochondrial function, demonstrated that mitochondrial dysfunction is a significant factor that accounts for impairments in aerobic capacity (r^2^ = 0.355), gait speed (r^2^ = 0.166), grip strength (r^2^ = 0.106), and even leg strength (r^2^ = 0.166) [45]. In fact, models of sarcopenia have demonstrated that mitochondrial dysfunction precedes the loss of redox homeostasis, the increase in oxidative damage to contractile proteins, and the decline in skeletal muscle function [53]. Thus, interventions that maintain and/or improve mitochondrial function will subsequently improve skeletal muscle function. We have previously highlighted the efficacy of targeting mitochondrial function to improve skeletal muscle proteostasis and function through improvements of energetics and the mitigation of oxidative damage [51,54]. In this review, we highlighted how mitohormetic responses elicited through aerobic exercise could resolve age-related impairments in redox homeostasis and mitochondrial function to maintain skeletal muscle function.

## 5. Redox Circuits and Redox Homeostasis

Reactive oxygen species (ROS), in acute and sublethal doses, are beneficial and confer health effects through a variety of mechanisms. However, there is an age-related increase in chronic ROS production which diminishes both stress resistance (prevention of a tipping point from adaptive to maladaptive response) and resilience (adaptive response and return to homeostasis) [55]. This unmitigated increase in ROS disrupts the redox signaling pathways necessary to defend against and adapt to oxidative challenges. As a consequence, unmitigated ROS causes oxidative damage of cellular components, including proteins and lipid membranes [56]. Age-related increases in oxidative stress disrupt redox homeostasis (i.e., the culmination of redox signaling circuits involved in sensing, signaling, and adapting to a stress) and promote the accumulation of oxidative damage both of which eventually lead to cellular dysfunction in muscle and other tissues. As a consequence, impaired redox signaling and oxidative damage predispose aged individuals to chronic diseases such as diabetes, Alzheimer’s disease, and sarcopenia [57]. Increased ROS production in skeletal muscle leads to the oxidative damage of proteins, such as contractile proteins, which compromises proteome integrity [49,58]. Moreover, oxidative damage to mitochondria impairs their capacity to generate ATP [59], which in turn impairs processes critical to maintaining the proteome (proteostasis) and myocellular function [53]. Thus, targeting redox homeostasis and mitigating oxidative damage have the potential to improve skeletal muscle function.

**Figure 1:**
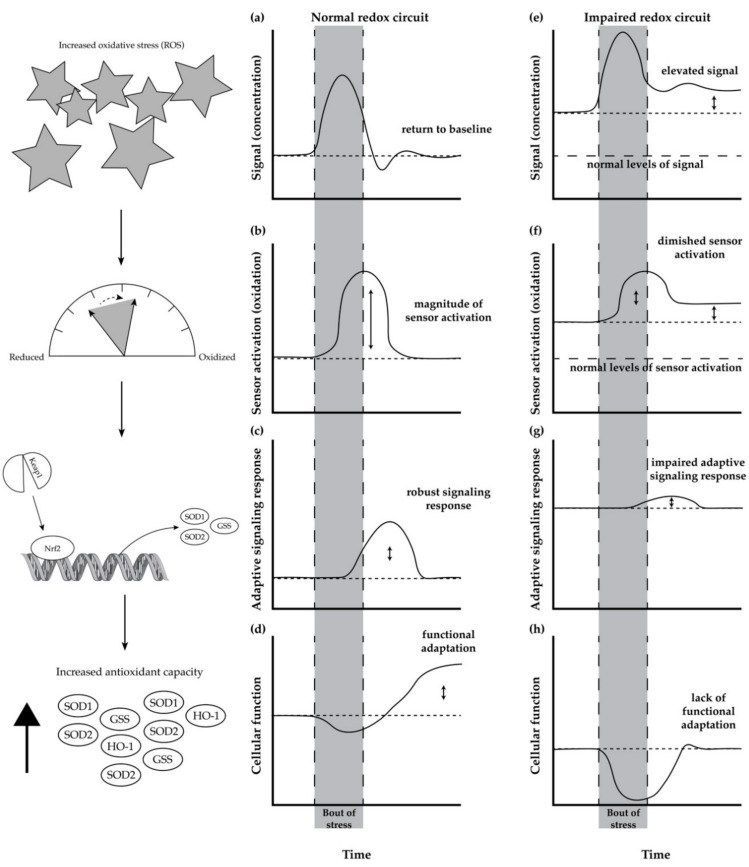
A normal and impaired redox circuit responding to acute stress. At the onset of a stress, the signal (e.g., ROS) increases (**a**), which causes activation of its respective sensor (**b**). In turn, an adaptive response pathway is activated (**c**), which elicits an improvement in cellular function (**d**) resolving both the stress signal and modified sensor to pre-stress levels. In addition, the adaptive response triggers improved function that is sustained well after the stress (**d**). With age, however, resting stress levels are elevated (**e**), which leads to greater signal stimulation pre-stress (**f**). However, the adaptive mechanisms are impaired and desensitized, such that these elevated levels of stress are not lowered. Consequentially, upon stress of a similar magnitude (**e**), the magnitude of sensor activation is smaller (**f**), which limits the adaptive response to the stress (**g**). As a result, the diminished response fails to improve functional capacity in response to a stress (**h**). In some cases, in an impaired redox circuit, the acute stress impairs cellular function. The left column shows an example of a redox circuit involving the Nrf2/Keap1 response pathway.

Redox homeostasis, the maintenance of many redox circuits, is impaired with age. In its simplistic form (Figure 1a–d), a redox circuit is comprised of a signal (Figure 1a), a redox sensor (Figure 1b), the activation of a response pathway (Figure 1c), and the functional outcome of the response (Figure 1d) [55]. In a broad sense, a bout of stress leads to an increase in a stress signal (indicated by an increase in the y-axis of Figure 1a), which then leads to the modification of a sensor to an activated state (increase in y-axis of Figure 1b). The activation of the sensor leads to the stimulation of a response pathway (Figure 1c), which then leads to functional improvements (Figure 1d).

To serve as an example of a redox circuit, we will walk through a redox circuit involving the Nrf2 (nuclear factor erythroid 2-related factor 2) signaling response pathway, which is responsible for the expression of antioxidant enzymes in response to ROS and other stressors. The redox circuit functions in this manner: hydrogen peroxide (Figure 1a, the signal) oxidizes cysteine residues (Figure 1b, the sensor) of the Nrf2/Keap1 complex, which leads to activation of the transcription factor Nrf2 (Figure 1c, the response pathway), and an increase in cellular antioxidant enzymatic capacity (Figure 1d, the functional outcome) [60]. The increased antioxidant capacity of the cell subsequently counterbalances the elevated production of ROS (return to baseline in Figure 1a), and it improves the overall function (elevated function in Figure 1d).

### Age-Related Impairment in Redox Homeostasis and Its Consequence in Skeletal Muscle

With age, in a seemingly paradoxical manner, there is an increase in baseline antioxidant enzyme expression (Figure 1g, response pathway) in response to unmitigated elevated levels of ROS (Figure 1e, signal) and increased cysteine oxidation (Figure 1f, sensor). However, due to desensitization to the signal and increased sensor activation [55], the impaired redox circuit fails to elicit a functional adaptation or enhance antioxidant capacity (Figure 1h) [61]. This is partially due to the fact that Nrf2 activity decreases with age [62,63]. In some cases, the stress fails to improve or, instead, impairs function. As a consequence, because there is no functional adaptation, the increased concentration of ROS is unresolved and instead it progressively increases (Figure 1e).

While there is still much to elucidate regarding the desensitization of redox circuitry, a modeling paper published in 2018 suggested that persistently elevated levels of stress or inflammation lead to “molecular habituation”, which leads to the desensitization of essential adaptive pathways [55]. Elevated levels of ROS (the signal) impair redox signaling by disrupting the initiation (via a sensor) of an adaptive response to the stress, thereby impairing the ability to functionally adapt to the stress [64,65]. Aged individuals have higher resting levels of ROS production, and they also have an impaired ability to acutely increase ROS to elicit an adaptive response [62,66].

As an example, glutathione (GSH) and oxidized glutathione (GSSG) are an essential redox (sensor) pair that helps to buffer increases in oxidants and activate adaptive responses to stress. As the ratio between GSSG/GSH shifts towards a greater oxidized state (i.e., the ratio increases), the sensor stimulates adaptive mechanisms. However, with age, the impaired ability to resolve oxidative stress [62,64] (Figure 1a→Figure 1e) leads to the constitutive oxidation (signal) of the GSSG/GSH redox pair (Figure 1b→Figure 1f) [61,67]. In this case, elevated GSSG/GSH constantly attempts to stimulate pathways to mitigate the stress; however, the capacity to respond to the signaling is also diminished with age (Figure 1c,d→Figure 1g,h). The impaired adaptive response (e.g., decline in the Nrf2 signaling response) to a stress would lead to a decline in the production of enzymes, such as glutathione synthetase and glutathione reductase, which are responsible for the maintenance of GSSG/GSH redox homeostasis. As a result, the ability to restore the GSSG/GSH ratio (sensor) to a normal redox state (i.e., a more reduced state), so that it can adequately detect changes in ROS (signal) and elicit a response (adaptation), is impaired [67].

It is important to note that the redox signaling pathway involving Nrf2 is just one example of an age-related impairment in a discrete redox circuit. There are a multitude of other redox circuits, only a few of which we address, that contribute to overall redox homeostasis (e.g., the unfolded protein response (UPR) [68]; the protein phosphorylome in response to redox modulation [69]) and have different roles than the Nrf2 signaling pathway in maintaining redox and protein homeostasis. These redox circuits all share a similar theme in that progressive, age-related disruption leads to dysfunction of the circuit and consequentially leads to decrements in function.

In aged skeletal muscle, because redox circuitry is impaired, the ability to resolve oxidative stress is diminished. It is likely there are subsequent effects that lead to skeletal muscle dysfunction with age. From an energetic perspective, excessive oxidative stress and oxidative damage contribute to mitochondrial dysfunction, which likely impairs the ability of the muscle to generate sufficient ATP for muscle contractions [70]. Unmitigated and sustained increases in ROS also damages proteins, including proteins responsible for contractions [48,71]. Mitochondrial dysfunction also impairs the UPR, thereby hindering a proteostatic mechanism to repair damaged proteins and maintain proteome integrity [72]. The accumulation of damaged proteins impairs the contractile machinery responsible for force production and transduction [41,73,74]. Disruption of the redox homeostasis also deleteriously affects muscle regeneration and skeletal muscle precursor differentiation [75]. In all, the unmitigated, elevated levels of ROS, combined with impaired redox signaling, lead to the accumulation of damaged cellular components and muscle dysfunction. Therefore, efforts to maintain or repair redox signaling with age are necessary to restore skeletal muscle function.

## 6. Mitohormesis as a Mechanism to Restore Redox Homeostasis

The term mitohormesis was first proposed in 2006 referring to the notion that sublethal mitochondrial stress can stimulate a robust cellular response that improves mitochondrial and overall cellular function [76]. For example, acute exposure to an oxidative stress can elicit a mitohormetic response characterized by improved protein folding and prevention of the age-related collapse of proteome integrity [77]. Mitohormesis is likely a critical mechanism that contributes to healthspan extension. For example, sublethal stresses that extend lifespan and health, such as caloric restriction or rapamycin treatment, also improve mitochondrial function mediated partially through increased mitochondrial turnover [78,79,80]. Mitohormesis likely contributes to health benefits by upregulating antioxidant enzymes [81], increasing mitochondrial biogenesis [79,81], enhancing mitochondrial function [82], and improving redox homeostasis [81]. To date, aerobic exercise training is one of the best examples of mitohormesis, where repeated acute bouts of stress elicit beneficial effects on health and function [83].

### 6.1. Mitohormesis, Aerobic Exercise, and Healthspan Extension

**Figure 2:**
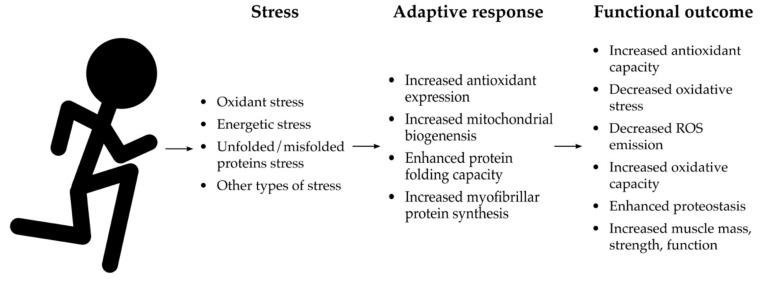
Mitohormetic effects of acute aerobic exercise. While there are broad effects of aerobic exercise that extend beyond mitohormesis, there are several mechanisms in which an acute stress improves health and skeletal muscle function. Through the stress response pathways indicated here, aerobic exercise stimulates myofibrillar protein synthesis, antioxidant expression, and mitochondrial biogenesis, as well as enhancing protein folding capacity. This translates broadly to improved redox homeostasis, mitochondrial function, and proteostasis. Altogether, these adaptive responses translate to enhanced skeletal muscle health and function.

Aerobic exercise has seemingly innumerable benefits on overall health that are well-documented in comprehensive reviews [84,85,86,87,88]. Perhaps one of the most potent effects of aerobic exercise training is on cardiorespiratory fitness (i.e., VO_2_max) [89]. Cardiorespiratory fitness is one of the strongest predictors of mortality. Given that individuals with higher cardiorespiratory fitness live longer than those with average or below average cardiorespiratory fitness, aerobic exercise training is one of the few established healthspan extending interventions practiced in humans to date [8,90,91,92]. In skeletal muscle, aerobic exercise training enhances mitochondrial function [93,94], stimulates skeletal muscle hypertrophy [88], and maintains strength and function throughout life [95,96]. Aerobic exercise protects from age-related chronic diseases such as Alzheimer’s disease [86], cancer [97], cardiovascular disease [98], diabetes [99], and many more [8]. These health benefits are conferred partially through mitohormetic effects of an aerobic exercise bout, where transient increases in ROS lead to cellular responses that protect skeletal muscle from damage and dysfunction (Figure 2).

### 6.2. Mitohormetic Effects of a Bout of Aerobic Exercise

The onset of exercise stimulates the generation of ROS from a variety of sources within skeletal muscle that is essential for muscle contraction [100]. For example, NADP(H) oxidases located in the sarcoplasmic reticular membranes, plasma membranes, and t-tubules, generate superoxide. The superoxide generated stimulates depolarization and calcium release necessary for muscle contraction [100]. Depleting ROS impairs force production whereas increased ROS production (up to the tipping point from adaptation to maladaptation) actually increases force production [101]. Therefore, ROS are necessary for adequate skeletal muscle function. In addition, a bout of aerobic exercise acutely imposes a cellular oxidative stress by transiently increasing ROS emission by simultaneously stimulating electron flux and decreasing ADP sensitivity [102]. It is important to point out that exercise-induced increases in ROS emission are transient, which is in contrast to the elevated, and often unmitigated, ROS production associated with aging. In fact, with sedentary aging, there is a diminished capacity to acutely elicit ROS emission during exercise [103], which impairs muscle contractile force and the adaptive redox circuits that impart cytoprotective effects [59,100,104]. However, aerobic exercise training seems to maintain and/or restore redox circuitry. 

At a molecular level, aerobic exercise acutely stimulates the generation of ROS and it activates a multitude of redox circuits related to stress adaptation [105] (e.g., AMPK [106], MAP kinases [107], and NFkB [108]) (Figure 2). ROS (the signal) also modify the activity of protein phosphatases (in this case, a sensor) that regulate the phosphorylation of proteins responsible for activating adaptive responses to muscle contraction [69]. Acute exercise also stimulates the UPR, another redox circuit, through increasing the protein folding demand [109,110]. Altogether, these redox circuits mediate the mitohormetic effect of acute exposure to stress imposed by a bout of exercise. For example, a bout of exercise stimulates Nrf2 activation in skeletal muscle which, as discussed, leads to the expression of antioxidant and other cytoprotective enzymes, as well as the enzymes responsible for improving mitochondrial and cellular function. The stimulation of AMPK through exercise-induced energetic and oxidative stress also stimulates mitochondrial biogenesis [111,112,113]. Acute activation of the UPR through exercise-induced oxidant stress also improves protein folding capacity and enhances the mechanisms related to proteostasis [114]. These examples of mitohormetic adaptation elicited through a bout of aerobic exercise highlight how aerobic exercise training extends the healthspan by maintaining skeletal muscle function and protecting against other age-related diseases (Figure 2). However, it is unclear whether age or redox dyshomeostasis blunts specific components of the adaptive response to a bout of aerobic exercise. Nonetheless, the benefits of aerobic exercise persist throughout the lifespan.

### 6.3. Mitohormetic Adaptations from Aerobic Exercise Training

Aerobic exercise training protects from age-related dysfunction and disease in a variety of ways that could be, at least in part, mediated through mitohormetic responses (Figure 2). For example, aerobic exercise training results in increased endogenous antioxidant defenses. ROS generated during exercise activate the transcription factor Nrf2, leading to the transcription of genes encoding much of the endogenous antioxidant network [115]. In turn, these enhanced endogenous antioxidant defenses mitigate the age-related increase in chronic oxidative stress and guard against dysfunction related oxidative damage [116]. There is also evidence to suggest that the activation of Nrf2 also has “crosstalk” promoting mitochondrial biogenesis [117]. Indeed, the stress imposed by exercise training has long been recognized as the most robust stimulus for mitochondrial biogenesis, increasingly recognized as redox-sensitive adaptation [102,118,119], resulting in enhanced mitochondrial function throughout age [120,121]. As a result of improved mitochondrial capacity, energetics seem to be more capable of investing in costly proteome maintenance, collectively called proteostatic mechanisms [51]. Aerobic exercise also increases myofibrillar protein synthesis to maintain the skeletal muscle contractile proteome [95] and contractile force [96], contributing to protection against age-related declines in strength and function. In summary, the mitohormetic response to aerobic exercise leads to cellular adaptations that translate to functional improvements that protect against age-related disease (e.g., sarcopenia) and extend healthspan.

## 7. Targeting Nrf2 as a Complementary or Alternative Approach to Restore Redox Homeostasis

Whilst exercise remains the most effective intervention to maintain and improve health, adherence to exercise guidelines remains remarkably low both in the United States (less than 10%) and Europe (less than 50%) [122,123]. Therefore, alternative and/or complementary therapies with better adherence rates could be utilized to maintain or increase healthspan. Given the detrimental role of age-related increases in chronic ROS production (as opposed to transient increases in ROS from a bout of exercise) on health, there has been emphasis on antioxidant supplementation to mitigate the age-related increases in oxidative stress to prevent, delay the onset of, and mitigate the severity of chronic diseases [124]. In this portion of the review, we highlight approaches that attempt to restore redox homeostasis and/or reduce oxidative stress.

### 7.1. Directly Scavenging ROS with Exogenous Antioxidants

Exogenous antioxidant supplements are compounds that can directly scavenge ROS, such as vitamin C and vitamin E. As oxidative stress emerged as a contributor to aging and disease, the potential for exogenous antioxidant supplementation as a means to mitigate excessive oxidative stress became a research focal point. However, several studies have demonstrated that exogenous antioxidant supplementation has deleterious effects on health and it may even block the mitohormetic effects of aerobic exercise.

Since exogenous antioxidants can directly scavenge ROS, they can also disrupt adaptive signaling pathways induced by ROS. This becomes apparent when observing the effects of supplementing aerobic exercise with vitamins C and E [125,126]. As discussed, aerobic exercise elicits an acute increase in ROS concentrations that stimulate redox signaling pathways, mitochondrial biogenesis, and other adaptive mechanisms that increase cardiorespiratory fitness [94]. However, supplementation with an exogenous antioxidant prevents the increase in ROS released from muscle contractile activity, which then abrogates the signaling pathways involved in the mitohormetic adaptation [125]. Beyond adaptation to exercise, a meta-analysis reveals that exogenous antioxidant supplementation provides no protection against chronic disease or mortality from chronic diseases [127]. This is likely due to the fact that exogenous antioxidants disrupt the redox signaling that is necessary for normal physiologic function and adaptation [128]. However, supplements that target endogenous antioxidant production to mitigate oxidant stress, whilst simultaneously allowing physiologic redox signaling to occur, may be more efficacious in eliciting beneficial adaptations and restoring redox homeostasis.

### 7.2. Upregulation of Endogenous Antioxidants

Aerobic exercise, as discussed, is a potent stimulator of endogenous antioxidant upregulation, resulting in the transcription of endogenous antioxidants, such as SOD1 and SOD2, which is mediated by the activation of transcription factors, such as Nrf2 [70,116,129]. As opposed to exogenous antioxidant supplements, there are compounds, which are often comprised of phytochemical components, that enhance cellular antioxidant capacity by upregulating the expression of antioxidant enzymes, such as SOD1 and catalase. As discussed, exogenous antioxidant supplements seem to abrogate important redox signaling leading to beneficial adaptations, because they directly scavenge oxidants. In contrast, enhancing endogenous antioxidant capacity permits redox signaling whilst simultaneously preventing ROS from reaching a tipping point in which a stress becomes maladaptive [130].

Recent research has demonstrated that the upregulation of endogenous antioxidants has beneficial effects on skeletal muscle function and overall organismal health. Phytochemical Nrf2 activators are one approach used to upregulate endogenous antioxidants. Nrf2 activators stimulate the translocation of Nrf2 into the nucleus leading to the transcription of the endogenous antioxidant genome [60]. As opposed to exogenous antioxidant supplements, Protandim, an Nrf2 activator, has been shown to extend the median lifespan in male heterogenous mice [131]. Our lab has demonstrated that treatment with Protandim protects coronary endothelial cells and cardiomyocytes from oxidative stress challenges [132,133]. Again, in contrast to exogenous antioxidant supplements, treatment with Protandim also enhanced proteostatic mechanisms and permitted the mitohormetic adaptations to physical activity [134]. Finally, our lab has demonstrated that treatment with a similar phytochemical Nrf2 activator enhanced the proteostatic maintenance of skeletal muscle contractile proteins in sedentary, healthy older adults [135]. Together, these findings suggest that Nrf2 activators may improve skeletal muscle quality and help maintain muscle function with age. Other Nrf2 activators, such as sulforaphane, demonstrate similar results, including improved mitochondrial and skeletal muscle function [136,137,138]. Moreover, other compounds that improve redox homeostasis through other mechanisms also seem to enhance skeletal muscle function. For example, SS31 is a peptide that protects cardiolipin, a phospholipid necessary for the maintenance of mitochondrial supercomplexes, and it improves mitochondrial function by increasing energetic capacity and reducing aberrant generation of mitochondrial ROS. Treatment with SS31 in old mice results in improvements mitochondrial function, redox homeostasis, and skeletal muscle function [139,140]. Thus, improving redox homeostasis, either by enhancing endogenous antioxidant capacity or decreasing ROS emission, appears to be a promising target to maintain skeletal muscle homeostasis throughout age.

## 8. Gaps and Future Directions

While the study of aerobic exercise on health and aging is decades old, the paradigm in which aerobic exercise exerts a mitohormetic effect is relatively new [83]. Given the multitude of effects of aerobic exercise [141], it is difficult to isolate the specific set of mechanisms by which aerobic exercise improves redox signaling and homeostasis. The relationship between exercise intensity/duration and the adaptive response to that bout, or to a series of bouts, remains unclear. There is particular difficulty in measuring the magnitude of stress that a single bout of exercise imposes and the magnitude of the mitohormetic effect. Establishing this relationship is further complicated when tailoring a bout of aerobic exercise for a given individual’s preexisting health, fitness level, level of redox homeostasis, and age. Measuring the degree of oxidation of known redox pairs (e.g., a sensor like glutathione/oxidized glutathione) or the protein phosphorylation (another sensor) before and immediately after exercise may assist in establishing the link between exercise intensity and the mitohormetic effect. However, the invasive nature of these measures (i.e., the requirement of multiple biopsies) and the meticulous adherence to tissue harvesting and treatment required to accurately measure these effects are quite prohibitive [69,142].

While both adaptation and the health benefits of both acute and long-term aerobic exercise are well understood, it is less clear how the mitohormetic response of a single bout of aerobic exercise changes with prolonged training. Given that aerobic exercise training increases antioxidant enzymatic capacity, does the mitohormetic effect of the same bout of exercise diminish with repeated bouts? Does the amount of ROS necessary to elicit the same mitohormetic effect increase as training progresses? In that context, questions arise as to whether there is a point where aerobic exercise training has a minimal or negligible mitohormetic effect, and it is instead necessary to maintain redox homeostasis.

Finally, work is required on the efficacy of increasing endogenous antioxidant capacity to enhance the mitohormetic effect of exercise, as well as to maintain or improve redox signaling with age. As highlighted, exogenous antioxidant supplementation seems to abrogate many of the mitohormetic effects of exercise. However, there appears to be some additive effect of improving cellular antioxidant capacity to exercise, as demonstrated by our work in which Nrf2 activators enhanced mitochondrial proteostasis in active rats [134] and increased proteostatic mechanisms in the skeletal muscle of older adults [135]. However, it remains unclear how a Nrf2 activator affects redox homeostasis. One would hypothesize that this may decrease the resting ROS concentration (signal), improve sensitivity to a stress (sensor), and enhance the adaptive response and functional outcome to that stress. However, these questions remain unanswered. Moreover, there is a lack of evidence on whether long-term treatment with Nrf2 could prevent or delay the onset of age-related redox derangements. Thus, further investigation is required into the field of targeting redox dysregulation to maintain skeletal muscle function with an Nrf2 activator. Moreover, it is necessary to conduct further research into how Nrf2 activators interact with aerobic exercise, a known mitohormetic intervention that restores redox homeostasis.

## 9. Conclusions

Extending healthspan will decrease the health and economic burden of age-related chronic diseases such as sarcopenia and improve quality of life throughout the aging process. Targeting the mechanisms that are characteristic of age (i.e., the hallmarks of aging) presents an opportunity to mitigate age-related disease and extend the healthspan. Redox homeostasis, which is comprised of multiple redox circuits, involves sensing and adapting to stress. With age, redox homeostasis is impaired, leading to oxidative damage of mitochondria and skeletal muscle dysfunction. Mitohormesis is a mechanism that elicits beneficial adaptation that can restore redox homeostasis and protect skeletal muscle from mitochondrial dysfunction and oxidative damage. A bout of aerobic exercise exerts a mitohormetic effect that results in the maintenance and improvement in mitochondrial function, antioxidant capacity, and proteostatic mechanisms (Figure 2). In turn, aerobic exercise training prevents the age-related decline in skeletal muscle function and extends the healthspan. Future directions should continue to elucidate the mechanisms in which exercise confers adaptation and restores homeostasis. Additionally, alternative or complementary interventions are necessary to maintain or restore redox homeostasis as a means to maintain skeletal muscle function and healthspan.
